# Stressors Length and the Habituation Effect—An EEG Study

**DOI:** 10.3390/s22186862

**Published:** 2022-09-10

**Authors:** Izabela Rejer, Daniel Wacewicz, Mateusz Schab, Bartosz Romanowski, Kacper Łukasiewicz, Michał Maciaszczyk

**Affiliations:** Department of Computer Science and Information Technology, West Pomeranian University of Technology in Szczecin, 70-310 Szczecin, Poland

**Keywords:** EEG signal, stress, habituation, repeated stimuli, stimulus length

## Abstract

The research described in this paper aimed to determine whether people respond differently to short and long stimuli and whether stress stimuli repeated over time evoke a habituation effect. To meet this goal, we performed a cognitive experiment with eight subjects. During this experiment, the subjects were presented with two trays of stress-inducing stimuli (different in length) interlaced with the main tasks. The mean beta power calculated from the EEG signal recorded from the two prefrontal electrodes (Fp1 and Fp2) was used as a stress index. The main results are as follows: (i) we confirmed the previous finding that beta power assessed from the EEG signal recorded from prefrontal electrodes is significantly higher for the STRESS condition compared to NON-STRESS condition; (ii) we found a significant difference in beta power between STRESS conditions that differed in length—the beta power was four times higher for short, compared to long, stress-inducing stimuli; (iii) we did not find enough evidence to confirm (or reject) the hypothesis that stress stimuli repeated over time evoke the habituation effect; although the general trends aggregated over subjects and stressors were negative, their slopes were not statistically significant; moreover, there was no agreement among subjects with respect to the slope of individual trends.

## 1. Introduction

Stress is an inevitable part of life. Regardless of age, gender or place of residence, humans experience tensions associated with various situations. These tensions manifest themselves in many different ways. Some people react with excessive sweating and others with acid stomach, backache, muscle tension, headaches, or rapid heartbeat [1,2]. Sudden emotional stresses can also trigger heart attacks, arrhythmias, and even sudden death [3]. Stress is a highly personalised phenomenon that varies depending on individual vulnerability and resilience [4].

The term stress is inconsistently used in the scientific literature. It may refer to a stimulus, a response to a stimulus or the physiological consequences of that response [5]. One of the definitions of psychological stress widely accepted in the field defines stress as a particular relationship between the person and the environment that the person regards as taxing or exceeding his or her resources and endangering his or her well-being [6]. Stress can affect a person positively or negatively; in other words, it can facilitate as well as impair performance [7]. On the one hand, stress may motivate a person to perform better, increasing their effectiveness simultaneously. On the other hand, stress can deteriorate the effectiveness of a task, and in extreme cases, it can even paralyse a person completely. It can sometimes be severely damaging or traumatic, leaving the developing individual permanently impaired, for example, by forcing withdrawal from life activities [7].

Psychologists mention the following two types of stress: acute and chronic. While acute stress is the result of demands and pressures of the recent past as well as those anticipated in the near future, chronic stress is due to long-standing pressures and demands, including those experienced as a result of socioeconomic conditions, difficulties in interpersonal relationships or an unsatisfying career [8]. Acute stress can emerge in situations such as test taking, meeting new people, or providing a speech in front of a large audience or can be evoked by a sudden unexpected event, unpleasant music or time pressure. In other words, it is triggered by episodic stressors, i.e., discrete events with a beginning and ending [9]. Stress can therefore appear suddenly as a result of a current situation or can accumulate gradually throughout a daily routine. At the same time, it can disappear quickly or can last for an extended period. Some people become used to stressful situations, while others do not. However, for both types of people, it might have a negative impact on health or even lead to illnesses such as depression [9].

Since different people react to stress differently, it is difficult to monitor stress levels accurately. In the past, a number of questionnaires were developed to measure stress perceived by individuals [10], such as Cohens’s Perceived Stress Scale (PSS) [11,12], Stress Response Inventory (SRI) [13], and Hamilton Depression Rating Scale (HDRS) [14]. Those psychological questionnaires are widely used but are also criticised since they introduce subjective bias into stress assessment. However, since stress presents itself via biomarkers and physical expression [8], it can be measured not only subjectively but also objectively by measuring physical reactions and physiological changes [15]. The methods that assess stress levels by analysing physical reactions typically focus on observing changes in behaviour, body gestures [16], speech [17], eye activity [18,19,20] or facial expression [21,22]. On the other hand, methods from the second group measure the reactions not visible from the outside, i.e., reactions such as changes in cortisol level [21], heart activity [23], brain activity [24,25,26,27,28,29,30,31,32], muscle activity [33,34], blood activity [35,36], respiratory response [37] or sweating response [38,39].

Since the stress mechanism begins in the brain [15], the stress level is often assessed through direct monitoring of brain activity, either with neuroimaging techniques such as fMRI (functional Magnetic Resonance Imaging) or techniques that record temporal changes such as fNIRS (Functional Near-infrared Spectroscopy) or EEG (Electroencephalography). Among those three, EEG is often chosen due to its long and well-established history and excellent temporal resolution, which is essential for measuring the development of stress response over time. Although the EEG technique cannot provide complete insight into the two of the three brain regions recognised as the most important for building the stress response (the hippocampus and amygdala) [40], the third region, the prefrontal cortex, is suited to this technique.

EEG measures the electric fields generated by the sum of the momentary post-synaptic potentials in the brain [41]. The EEG signal recorded in time by electrodes located over different brain areas is usually analysed either directly in the time domain by assessing the amplitude and latency of the peaks and troughs of event-related potentials (ERPs) or after transformation to the frequency domain by assessing the power of rhythms in some specific frequency bands. Most stress analysis studies adopt the second of the mentioned approaches and assess the changes in stress level via alterations in power in specific frequency bands over specific brain regions. Traditionally, the whole EEG frequency band is divided into the following five smaller bands: delta (1–4 Hz), theta (4–8 Hz), alpha (8–13 Hz), beta (13–30 Hz), and gamma > 30 Hz. Out of all those bands, alpha and beta bands (and their combinations) are usually used to study responses to stress stimuli.

Although there are some conflicting results in spectral features analysed in stress studies, stress conditions are considered to decrease the alpha activity and increase the beta activity [15]. While beta power indicates that a human is in an alertness condition, alpha power is usually related to the relaxation condition [27]. Many independent studies have confirmed these two tendencies. For example, [28] reports a significant decrease in alpha rhythm power (*p* < 0.01) and an increase in beta rhythm power (*p* < 0.02) in the prefrontal cortex (PFC) under the stress condition. Similar results were shown in [42], where it was found that the alpha/beta and theta/beta ratios are negatively correlated with stress. Further, in [27], it was reported that the high-stress group (the group performing the IQ test) featured less alpha and more beta energy over the PFC than the three control groups (performing no task at all). On the other hand, in [43], where the effect of an indoor environment on stress level was analysed, higher relative high-beta power over temporal brain areas was reported for the stress condition (temperature 30 °C, odour irritants, traffic noises) compared to the relaxed condition (temperature 25 °C, non air pollutant, nature sounds).

Habituation is traditionally defined as a behavioural response decrement that results from repeated stimulation and that does not involve sensory or motor adaptation or fatigue [44,45]. This process occurs in the nervous system of all living organisms. All behavioural responses, as well as autonomic and EEG responses and signs of arousal, habituate rapidly and to a profound degree. Behavioural responses that undergo habituation may include any final output of the nervous system, including pupillary responses, sweating, muscle contraction, hormone release, molecular responses, or neuronal activity, including population activity measured with EEG or functional imaging [44].

One of the first studies, where the habituation phenomenon was analysed via the changes in EEG signal, was that performed by Sharpless and Jasper [46]. Using repeated stimulation with brief tones, they found that cortical EEG arousal of sleeping cats becomes progressively shorter, and with time it disappears entirely. After stopping the stimulation, the arousal response recovers to the initial value, although it may take some time, ranging from several minutes to hours [47]. Four years later, Sokolov [48] showed that the human alpha blocking response—a human equivalent of EEG arousal in cats—habituates tactile, auditory and visual stimulation.

Habituation to repeated stress stimuli has been analysed in terms of different physiological and behavioural responses. The approach that is taken most often in stress habituation studies is that of hormonal habituation [49,50,51]. Among others, approaches analysing blood pressure and heart rate [52], blood coagulation parameters [53], or cardiovascular reactivity [54] are also reported. Although the research on EEG tracks of habituation started a long time ago, very few studies analyse alterations of EEG signals in terms of repeated stress stimuli. In this paper, we aim to contribute to this domain and analyse the electrical brain response to a stress stimulus repeated in time.

The research described in this paper focuses on the following two main questions: (i) Does the subject respond differently to short and long stimuli of the same type? (ii) Does the subject’s brain activity, measured with EEG electrodes, habituate to stress stimuli repeated over time? To shed light on both questions, we designed an experiment composed of two main tasks and carried it out with eight subjects. During the first task, the subject was reading the text, and during the second task, the subject solved a simple quiz composed of a set of single-choice questions. In both tasks, every 3–4 min, a pop-up window with a stress-inducing question appeared on the screen, interrupting the main task. The stress-inducing questions differed significantly in respect to their length, from precisely 5 s in the first task to 2 min (or less) in the second task. The stress was induced on the subjects via time pressure, unpleasant sounds, and flashing lights. The mean beta power calculated from the EEG signal recorded from the two prefrontal electrodes (Fp1 and Fp2) was used as a stress index.

The main results of the experiment were as follows: (i) we confirmed the previous finding that beta power assessed from the EEG signal recorded from prefrontal electrodes is a good predictor of stress response—in Task A, the beta power for the STRESS condition was almost three times higher than in the NON-STRESS condition; in Task B, this ratio was smaller but also statistically significant; (ii) we found a significant difference in beta power between stimuli that differed in length—the beta power was four times higher for Task A, where very short stimuli were used compared to in Task B, where significantly longer stressors were applied; (iii) we did not find enough evidence to confirm (or reject) the hypothesis that stress stimuli repeated in time evoke the habituation effect—although the general trends aggregated over subjects and stressors were negative, their slopes were not statistically significant either for Task A or Task B; moreover, there was no agreement among subjects in respect to the slope of individual trends.

This paper is structured as follows. Section 2 provides information on the experimental setup, data collection process, and the procedures used for EEG signal processing. Section 3 presents all the results together with their statistical analysis. Finally, Section 4 provides the interpretation of the findings reported in the paper.

## 2. Materials and Methods

An experiment with eight healthy subjects (students of Computer Science) was conducted to test the subjects’ response to stress-inducing stimuli of different length. All subjects were right-handed and reported no mental disorders. The subjects’ demography is presented in Table 1. The experiment was conducted under the permission obtained from the Bioethical Committee of the Medical Chamber in Szczecin (OIL-Sz/PH/KB/452/01/04/2020) and in accordance with relevant guidelines and regulations stated in the Declaration of Helsinki. Informed consent was obtained from all subjects. Before the onset of the experiment, each subject was introduced to its basic structure.

The experiment was conducted as a competition to motivate the subjects to higher engagement and increase their stress level. The subjects were informed that 50% of the best performing participants would be rewarded at the end of the experiment. Each subject participated in the experiment individually without communication with others, and his performance was evaluated based on the number of correct answers.

The experiment was composed of two main tasks, Task A (reading comprehension task) and Task B (basic knowledge single choice quiz). Both tasks started with a short calibration phase divided into two stages. The first stage aimed to relax a participant before the main task. During this stage, a subject was asked to listen to a piece of anti-stress music (with eyes opened). After thirty seconds, the relaxation stage was finished and the participant performed a pseudo-Stroop number test. During the test, the pairs of different numbers were displayed on the computer screen. Both numbers from each pair differed significantly in the font size. The subject’s task was to choose a number that was greater in value. The time allowed for providing an answer was limited to 800 ms. When a subject chose a wrong number or the time ran out, there was a loud ’error’ noise and the screen flashed for 2 s to instruct the subject that a mistake was made. After five mistakes, the test ended and the main part of the experiment started. The order of tasks (Task A and Task B) was chosen randomly for each participant to ensure that it did not influence the stress level.

**Figure 1:**
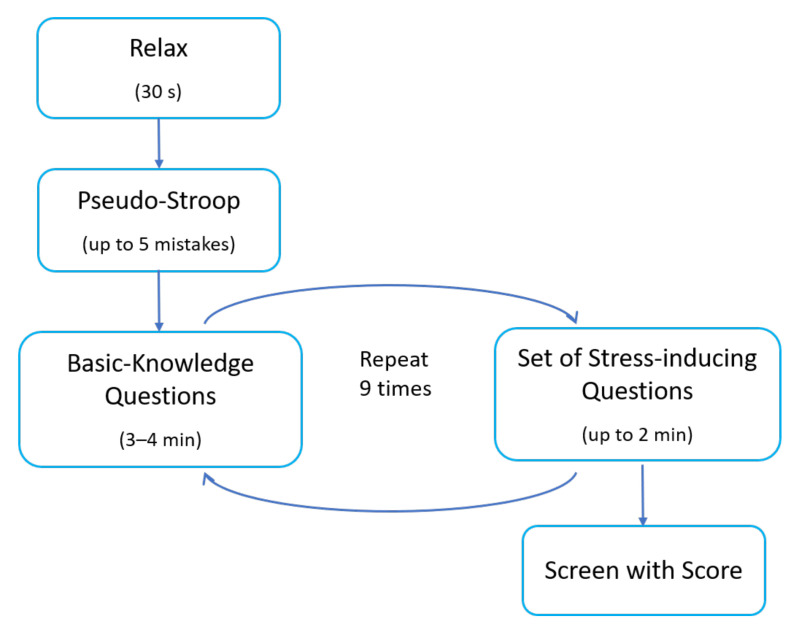
The order of events in Task A.

In the reading comprehension task (Task A), the participant read a text displayed on the computer screen. The text conveyed an exciting but quite dark and mysterious story. When building the creepy atmosphere, quiet, peaceful music was played in the background. When the reader reached a certain point in the text, a window with a single multiple choice question regarding the previously read part of the text appeared in the central part of the screen. The subject had only five seconds to read the question and the possible answers and choose one of them. After that time, the window disappeared from the screen, and the subject could return to the reading task. The pop-up window was meant to induce stress in participants in the following four ways: a sudden change in the screen, flashing colours inside the window, a change in music - from quiet and peaceful to loud and unpleasant, and a large stopper showing the running time. During the whole task, ten stress-inducing questions were displayed for each participant. The order of events in Task A is presented in Figure 1.

**Figure 2:**
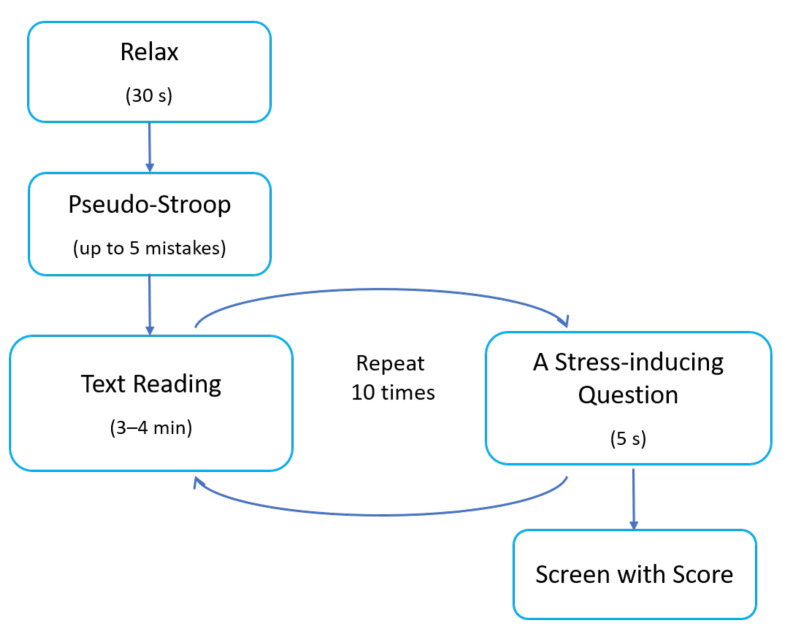
The order of events in Task B.

Task B was a single-choice quiz composed of questions testing the subjects’ common knowledge. The questions, with four possible answers, were displayed one at a time on the computer screen. The subjects finalised their answer by clicking on the ’Next question’ button displayed at the bottom of the screen. The time given for answering each question was unlimited. Every 3–4 min, a window with a stress-inducing question was triggered. Questions of that type were composed of many stages and were limited in time to up to two minutes. They also appeared on the screen as pop-up windows (similar to Task A) and were accompanied by loud music, flashing colours inside the window, and a large stopper showing the running time. There were two types of stress-inducing questions: questions asking the subject to choose a correct category for a given object and questions asking the subject to order objects correctly. Each stress-inducing question was composed of a set of sub-questions reappearing in the window precisely after the subject finished answering the previous sub-question. The subject task was to answer all the sub-questions as quickly as possible but within the 2 min time limit. During the whole task, nine stress-inducing sets of questions were displayed for each participant. The order of events in Task B is presented in Figure 2.

The subjects’ brain activity was recorded with an EEG cap during the experiment. EEG data were collected from 19 monopolar channels at a sampling frequency of 500 Hz. The electrodes’ positions were set according to the International 10–20 system [55]. The ground electrode was attached at Fpz, and the reference electrode at a left mastoid. The impedance was kept below five kΩ for all electrodes. The signal was acquired with MITSAR 202 amplifier [56], recorded with Mitsar EEG Studio Acquisition software [57], and processed and analysed in a Matlab environment [58].

**Figure 3:**
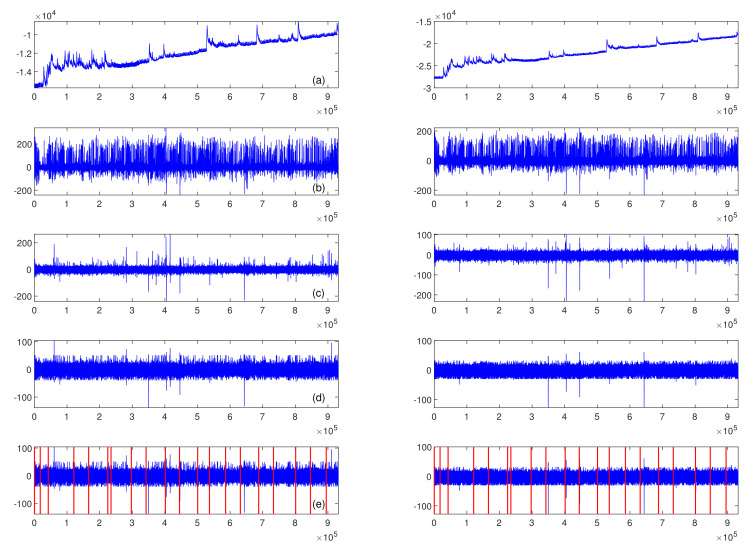
The preprocessing procedure via signal recorded from subject S1 performing Task B; left column of plots—Fp1 channel, right column of plots—Fp2 channel; (**a**) raw signal, (**b**) signal after temporal filtering, (**c**) signal after spatial filtering, (**d**) signal after median filtering, (**e**) signal divided into epochs, each vertical line corresponds to the epoch onset (the epochs’ order is presented in Figure 2).

A set of filters was applied to clean the EEG signal and prepare it for further analysis. First, a pair of Butterworth temporal filters of the 4th order was used to attenuate the signal outside the 0.5–40 Hz range, a high-pass filter with a cut-off frequency of 0.5 Hz and a low-pass filter with a cut-off frequency of 40 Hz. Next, a spatial filter was applied to remove EOG (electro-oculographic) artefacts. A Matlab implementation of the FastICA algorithm [59] was used to manage this task. The artefact components were identified based on the signal power in the 2–4 Hz frequency band. We removed components whose power exceeded the mean power calculated over all available components by three standard deviations. The remaining components were used to reconstruct channel signals from the two prefrontal electrodes, Fp1 and Fp2. Although the EEG signal was recorded from all 19 sides, only two channels were applied in further processing and analysis. The final filter used in the preprocessing stage was a median filter with a kernel length of 40 samples. The task of the median filter was to eliminate episodic spiking artefacts (outliers). The outliers’ thresholds were set to the 0.1 and 99.9 percentiles. Figure 3 presents the effect obtained after applying each of the mentioned filters on the signal recorded from Fp1 and Fp2 channels from subject S1 performing Task B.

The EEG signal was split into epochs when the processing stage was finished. Forty two epochs were extracted for each subject: (i) relax A, (ii) pseudo-Stroop test A, (iii) ten text reading epochs from Task A (non-stress condition), (iv) ten stress-inducing questions from Task A, (v) relax B, (vi) pseudo-Stroop test B, (vii) nine standard questions from Task B (non-stress condition), and (viii) nine stress-inducing questions from Task B. Next, the signal in each epoch was described by a single feature—a relative signal power in the beta frequency band (13–30 Hz). To obtain the relative power, the signal power in the beta band was divided by the total signal power, i.e., by the signal power in the whole 0.5–40 Hz band (Equation (1)).

(1) $$Power=\frac{Beta\;power}{Total\;power}*100\%$$

The relative signal power was calculated separately for the two prefrontal electrodes (Fp1 and Fp2). Next, both values were averaged to obtain one characteristic for each event. To align results across the subjects, the powers obtained for each subject from all 42 events were gathered together and normalised according to min-max normalisation (Equation (2)).

(2) $$PowerN_{i}=\frac{Power_{i}-min\left(Power\right)}{max(Power)-min(Power)}$$

where $Power_{i}$, $PowerN_{i}$—original/normalised power value of *i*-th event; max(Power), min(Power)—maximum/minimum power calculated over all events for a given subject.

## 3. Results

### 3.1. Stress vs. NON-STRESS Condition

We started the analysis by testing whether the subjects’ brain activity differed significantly between the STRESS and NON-STRESS condition. To this end, we performed two paired-samples tests. The first test tested the significance of beta power difference in Task A, and the second did the same for Task B. The significance level was set to 0.05 for these two tests and all other tests reported in this paper. Before running the tests, all four groups (STRESS and NON-STRESS for Task A and STRESS and NON-STRESS for Task B) were tested against the normality condition with a Lilliefors test. The Lilliefors tests did not provide evidence to reject the null hypothesis for the STRESS group in Task A but showed a significant deviation from normality for all of the other three groups (*p*-value < 0.05). Therefore, to test whether the STRESS and NON-STRESS conditions differed significantly in both tasks, we used a non-parametric paired-samples test by testing the medians difference, namely the Wilcoxon signed-rank test. To maintain consistency throughout the study, the same test was used in all further analyses, apart from the cases where the groups of different sizes were compared. In such cases, a non-parametric independent-sample test was used, namely the Wilcoxon rank sum test.

**Figure 4:**
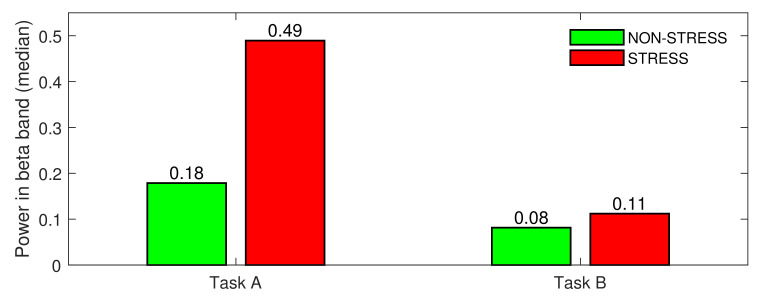
STRESS vs. NON-STRESS condition—difference in medians for Task A and Task B.

**Figure 5:**
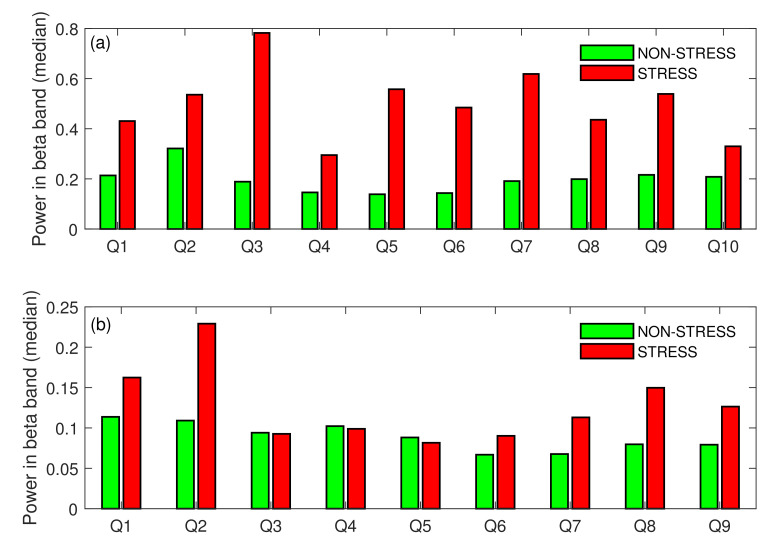
STRESS vs. NON-STRESS condition—difference in medians across events in Task A (**a**), and Task B (**b**); Q: Question.

The Wilcoxon signed rank test showed that the difference in medians calculated for the STRESS vs NON-STRESS condition was significant for both tasks (*p*-value = 0). As can be noticed in Figure 4, the difference in medians for Task A was very high (the beta power increased by 163% for the STRESS condition compared to the NON-STRESS condition) and much lower for Task A (36% increase in beta power was observed). When analysing each stress-inducing question individually, almost all agreed with this general tendency. Figure 5 presents the beta power (median over subjects) for each event from both main tasks. As can be noticed in the figure, the median of the beta power calculated over all stress-inducing events from Task A exceeded that calculated for non-stress-inducing events. Moreover, all of these differences were statistically significant (*p*-value < 0.05). In the case of Task B, most pairs of events also agreed with the general tendency, but there were three questions (Q3, Q4, Q5) where the medians were almost at the same level for both STRESS and NON-STRESS conditions.

**Figure 6:**
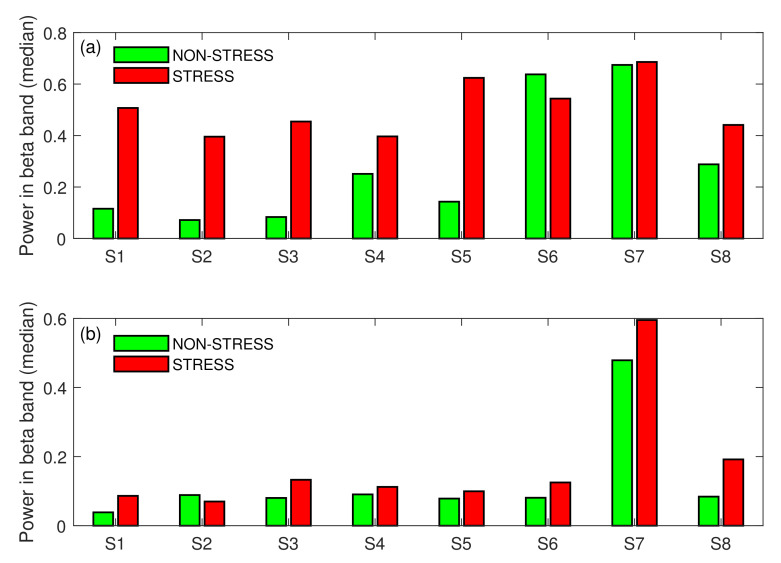
STRESS vs. NON-STRESS condition—difference in medians across subjects for Task A (**a**), and Task B (**b**); S: Subject.

Figure 6 shows that the tendency presented in Figure 4 was also stable across subjects. Only for subject S6 in Task A and subject S2 in Task B was the beta power slightly higher for the NON-STRESS condition, but these differences were insignificant (*p*-value > 0.05). For all other subjects in both tasks, the beta power in the STRESS condition was higher than in the NON-STRESS condition. Moreover, these differences were statistically significant (*p*-value < 0.05) for most of the subjects. Only for subject S7 were the differences in medians across conditions not significant (*p*-value > 0.05). Figure 4, Figure 5 and Figure 6 validate our experimental setup, i.e., they show that subjects were more stressed during answering pop-up questions than reading the text (in Task A) or during answering the stressful question than standard ones (in Task B). Hence, these figures set the ground for further analysis.

### 3.2. Short vs. Long Stimuli

**Figure 7:**
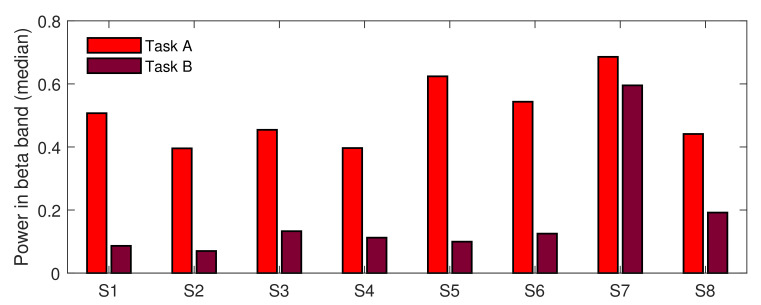
Task A vs. Task B—difference in medians across subjects for the STRESS condition; S: Subject.

The first of the two main goals of our paper was to test whether short stimuli are significantly more stressful than longer ones. To deal with this task, we performed another test, testing Task A against Task B under the STRESS condition. Because both groups’ sizes were unequal, we used the Wilcoxon rank sum test. The test showed that the difference in medians between both tasks was highly significant, with *p*-value = 0. To verify whether that result was consistent across subjects, we performed eight additional rank sum tests, testing task differences (under the STRESS condition) for each subject separately. Almost all tests (except the test performed for subject S7) provided evidence for rejecting the null hypothesis, testing the equality of medians (*p*-value < 0.05). Figure 7 presents the differences in medians across subjects. As can be noticed in the figure, the results are highly consistent; all medians calculated for Task A exceeded those calculated for Task B. This indicates that the short stimuli provided in Task A placed much higher pressure on subjects than longer stimuli provided in Task B. Of course, it should be mentioned here that not only the medians for the STRESS condition were higher for Task A. As shown in Figure 4, the general stress level during the whole of task A exceeded that of Task B. However, while in the non-stress-inducing parts of the tasks the stress level was only two times higher for Task A, the stress-inducing parts were four times more stressful in Task A than in Task B.

### 3.3. Stress over Time

**Figure 8:**
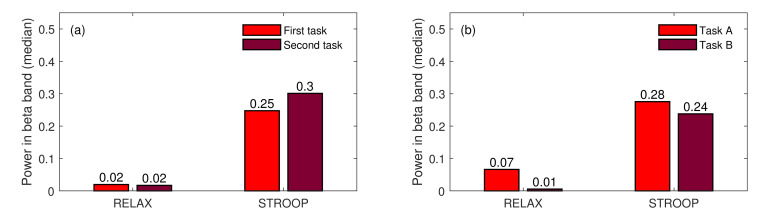
The comparison of medians for RELAX and STROOP events arranged in time (**a**), arranged according to tasks (**b**).

The second goal of our analysis was to determine whether subjects could adapt to the stress-inducing stimuli over time. We assumed that if we applied the same type of stimulus multiple times, the habituation phenomenon would be induced in the subject’s brain. We began the verification of this assumption by testing if there was any statistical difference in the pseudo-Stroop test performed at the beginning of the experiment and after finishing the second task. Since the order of the tasks was assigned randomly to each subject, we had to rearrange the data according to time. Figure 8 compares the medians calculated for STROOP (and RELAX) events in two layouts. The first two pairs of medians (in the first subplot) compare the events arranged in time (FIRST vs SECOND), and the second two pairs of medians compare the events arranged in tasks (Task A vs. Task B).

As can be noticed in the first subplot in Figure 8, the median calculated for the STROOP event was slightly higher for the second task than for the first one. However, this difference was not statistically significant (Wilcox signed rank test returned *p*-value equal to 0.3125). This difference could be caused just by the difference in STROOP events in both tasks (also statistically insignificant—*p*-value = 0.7422), which can be observed in the second subplot in Figure 8. Hence, the comparison of the two stress-inducing events did not show any habituation effect; rather, we observed the opposite with the second occurrence of the STROOP event placing slightly higher, instead of lower, pressure on the subjects.

**Figure 9:**
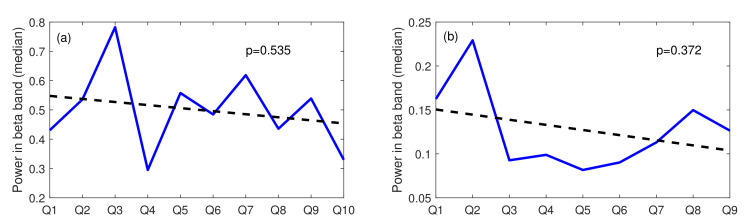
The stress-inducing events over time; (**a**) Task A, (**b**) Task B; Q: Question; blue line: beta power for each question, dotted line: linear trend.

The STROOP event occurred only twice during the whole experiment. This could be the reason for the lack of habituation in time. Therefore, to find out if more stress-inducing stimuli of the same type might evoke the habituation tendency, we analysed the stress-inducing events triggered during both main tasks. We started with task A, where subjects faced ten stress-inducing questions. We plotted the beta power calculated for each pop-up question to determine the tendency and evaluated a linear trend. We assumed that if a subject habituates to stress induced by the succeeding questions, the power values would be smaller with each question. Next, we did the same for task B. The trend lines obtained for both tasks are presented in Figure 9.

As shown in Figure 9, both trend lines have a negative slope, which might indicate the subjects’ habituation to the stress stimuli over time. Such a claim, however, is not fully justified since both trends are not statistically significant. A trend line is regarded as statistically significant when the *p*-value determined for the parameter indicating the line slope is smaller than the chosen significance level (set to 0.05 for all tests reported in the paper). This condition is not met either for Task A (*p*-value = 0.535) or for Task B (*p*-value = 0.372).

**Figure 10:**
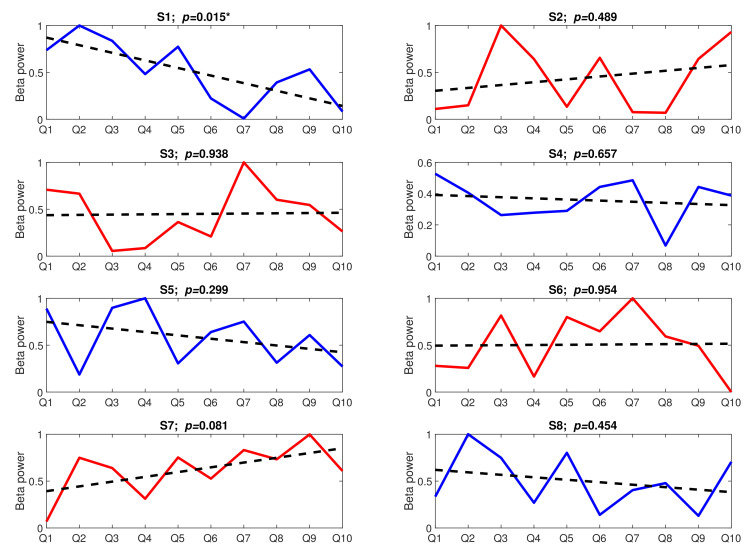
The stress-inducing events from Task A over time; upward trends are marked in red, downward trends are marked in blue, dotted lines present linear trends, asterisks (*) denote linear trends with a significant slope (*p*-value < 0.05); Q: Question, S: Subject.

**Figure 11:**
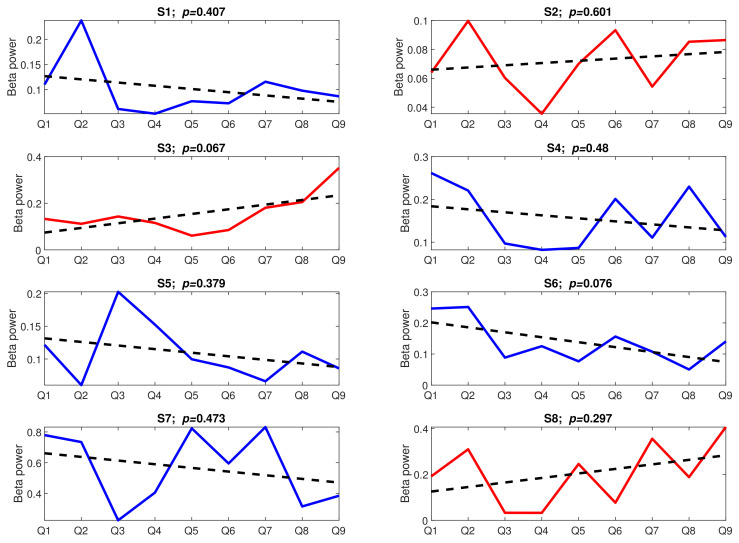
The stress-inducing events from Task B over time; upward trends are marked in red, downward trends are marked in blue, dotted lines present linear trends; Q: Question, S: Subject.

Figure 9 presents the trends aggregated over subjects. To look more thoroughly into the time effect, we analysed the trend lines in both tasks individually for each subject. The trend lines, together with their significance level, are presented in Figure 10 (Task A) and Figure 11 (Task B).

The results presented in Figure 10 and Figure 11 are very surprising. Not only are the trends not significant (*p*-value > 0.05) (apart from the trend found for subject S1 in Task A), but their slopes are even incoherent across subjects. In Task A, only four trend lines have a negative slope (S1, S4, S5, and S8), while the remaining ones have either a positive slope (S2, S3, and S7) or the slope is not visible at all (S6). The situation in Task B is similar; five trend lines have a negative slope (S1, S4, S5, S6, and S7), and the remaining three are positive.

## 4. Discussion

Previous research on EEG power features used to monitor stress level present somewhat contradictory findings [15]. For example, while in [27,28] the alpha activity was reported to be positively correlated with stress, in [60] the reverse pattern was claimed. Similarly, while in [27,28,43] the beta power was found to be higher for stress compared to relax condition, in [61] the correlation was the opposite. Therefore, although the primary goal of the research reported in the paper was to analyse the habituation phenomenon in response to stress stimuli, we started the research by testing whether the beta power measured from the prefrontal cortex is a good predictor of stress. As was shown in Section 3.1, the beta power calculated for both sequences of stress stimuli was significantly different from that calculated for the stimulus-free parts of the experiment (*p*-value < 0.05). In Task A, the beta power was almost three times higher for the STRESS condition compared to the NON-STRESS condition. In Task B, this ratio was smaller but also statistically significant. There were some deviations from this general tendency, e.g., for subject S6 in Task A and subject S2 in Task B or for three questions in Task B, but for most questions and subjects, the general tendency was held.

The main goal of the research reported in this paper was to analyse the habituation phenomenon in regard to stress. We did so by analysing the brain activity recorded from eight subjects exposed to two trains of stress stimuli. The stimuli used in both tasks of the experiment were very similar concerning the stress-inducing mechanisms. Both involved suddenly appearing pop-up windows, time pressure, flashing elements, and loud, unpleasant music. The two main differences between the stimuli used in Task A and Task B were their content and the time duration. As for the content, to balance the difficulty level of questions used in both tasks, we made them as easy as possible. We did not want the subject to become stressed by the question per se but rather by the running time and other stimuli appearing with the question. Hence, the real difference between both trains of stimuli was their length. While in Task A, the pop-up windows were present on the screen only for 5 s, in Task B, the subject had up to two minutes to answer all sub-questions reappearing in the window.

As shown in Figure 4, there was a huge difference between beta power calculated for both types of stress stimuli. While the median calculated over the stress-inducing questions and subjects for Task A was equal to 0.49, it was over four times lower (equal to 0.11) for Task B. In Figure 7, this general tendency was confirmed for all subjects participating in the experiment. Since the main difference between both tasks was the difference in stimulus length, we conclude that the difference in subjects’ brain activity should be attributed to the difference in stimulus length.

In Task B, subjects were exposed to the same stressors (time pressure, flashing lights, loud music) for up to two minutes. Their weaker response to those stimuli might result from the habituation phenomenon developing in time. Our experimental setup did not allow us to verify this hypothesis experimentally (due to the different lengths of each question and different times needed by each subject to answer all sub-questions within each question). However, it seems reasonable since the initial phase of stress-inducing events in both tasks was exactly the same. The difference was that the activity of stressors finished after five seconds in Task A and lasted up to 2 min in Task B.

Regarding habituation to repeated stressors, we did not confirm our preliminary hypothesis. At the beginning of the experiment, we expected that subjects would become used to the stress-inducing questions, which would be represented by the decrease in the prefrontal electrodes’ beta power with each consecutive question. Meanwhile, although both general trends calculated over all subjects (Figure 9) had an expected negative slope, they were not statistically significant (*p*-value > 0.05). Even less expected results can be observed in Figure 9 and Figure 10, where not only are the trends insignificant (*p*-value > 0.05), but also their slope is reversed for some subjects.

Comparing the last two figures (Figure 10 and Figure 11) along the subjects, one interesting feature can be noticed—for more than half of the subjects, the slopes of the trend lines agree across the tasks. For example, while for subjects S1, S4, and S5, the decreasing trend is observed in both tasks, the trend found for subjects S2 and S3 is positive. This might suggest that the adaptability to stress stimuli depends on the subjects’ general attitude to stress. While the habituation phenomenon might develop for some subjects when the stress stimuli are repeated over time, for others, each new stress stimulus might be regarded as more invasive than the previous one. Of course, since the slope of the trend lines is statistically insignificant for most subjects (*p*-value > 0.05), this is only a weak suggestion.

An intrinsic feature of the habituation phenomenon is that habituation is quicker and more visible in the case of a softer stimulus. As was stated in [44,45], the weaker the stimulus, the more rapid and/or more pronounced the habituation. Strong stimuli may yield no significant habituation. Our experiment did not show such a relationship for the two applied sequences of stress stimuli. Although there was a considerable disproportion between the beta power calculated for both tasks (Figure 4), there were no corresponding differences in trend slopes. Conversely, for Task B, where a weaker response was observed, more subjects showed signs of habituation (negative slopes of trend lines) than for Task A.

Stress is a very complex phenomenon. Many research confirmed that different people react differently to the same stressful situations [1,2,3,4]. Our research suggests that people show not only different physical or psychological reactions to stress-inducing stimuli but also different attitudes to stress stimuli repeated in time. While some might habituate to repeated stress stimuli (such as subjects S1, S4, S5, and S8 in Task A or subjects S1, S4, S5, S6, and S7 in Task B), others may react more strongly with each new stimulus (such as subjects S2, S3, and S7 in Task A or subjects S2, S3, and S8 in Task B).

The experiment reported in the paper was performed with eight subjects only. We used a small sample because we wanted to analyse not only the aggregated results but also the individual trends of each subject. Nevertheless, this small sample size is a limitation of our study because it did not provide enough evidence to form conclusions regarding the general population. Hence, the reported results should be considered as preliminary findings that require further investigation.

## Figures and Tables

**Table 1:** Subjects’ demography.

Subject	Age	Gender	Nationality
S1	22	Male	Polish
S2	24	Male	Polish
S3	24	Female	Polish
S4	22	Male	Polish
S5	24	Male	Polish
S6	23	Male	Polish
S7	22	Male	Ukraine
S8	23	Male	Polish

## Data Availability

The data presented in this study are available on request from the corresponding author. The data are not publicly available due to privacy restrictions.
